# Effect of Dataset Size and Train/Test Split Ratios in QSAR/QSPR Multiclass Classification

**DOI:** 10.3390/molecules26041111

**Published:** 2021-02-19

**Authors:** Anita Rácz, Dávid Bajusz, Károly Héberger

**Affiliations:** 1 Department of Plasma Chemistry, Institute of Materials and Environmental Chemistry, ELKH Research Centre for Natural Sciences, Magyar Tudósok krt. 2, H-1117 Budapest, Hungary; racz.anita@ttk.hu; 2 Medicinal Chemistry Research Group, ELKH Research Centre for Natural Sciences, Magyar Tudósok krt. 2, H-1117 Budapest, Hungary; bajusz.david@ttk.hu

**Keywords:** machine learning, XGBoost, validation, training/test split ratio, multiclass classification, imbalanced

## Abstract

Applied datasets can vary from a few hundred to thousands of samples in typical quantitative structure-activity/property (QSAR/QSPR) relationships and classification. However, the size of the datasets and the train/test split ratios can greatly affect the outcome of the models, and thus the classification performance itself. We compared several combinations of dataset sizes and split ratios with five different machine learning algorithms to find the differences or similarities and to select the best parameter settings in nonbinary (multiclass) classification. It is also known that the models are ranked differently according to the performance merit(s) used. Here, 25 performance parameters were calculated for each model, then factorial ANOVA was applied to compare the results. The results clearly show the differences not just between the applied machine learning algorithms but also between the dataset sizes and to a lesser extent the train/test split ratios. The XGBoost algorithm could outperform the others, even in multiclass modeling. The performance parameters reacted differently to the change of the sample set size; some of them were much more sensitive to this factor than the others. Moreover, significant differences could be detected between train/test split ratios as well, exerting a great effect on the test validation of our models.

## 1. Introduction

In the new century, the emphasis has shifted from mechanistic (explanatory) modeling to the widespread use of machine learning algorithms in QSAR and related fields as well. In statistics, the long-term commitment to data models only (i.e., with the assumption of a stochastic model) “led to irrelevant theory, questionable conclusions, and has kept statisticians from working on a large range of interesting current problems” [1]. In the meantime, algorithmic modeling has developed rapidly and found its way to application domains formerly employing classic statistical tools, such as QSAR or drug design in general. Especially for larger dataset sizes, machine learning tools present much more suitable alternatives for classification than conventional statistics.

While drug design applications routinely employ two-class classification tasks (i.e., machine learning models for predicting active/inactive compounds), multiclass classification scenarios are somewhat less common and, accordingly, less studied. This is also reflected in the number of available performance parameters for two- vs. multiclass classification, although some of the performance metrics naturally extend to the multiclass case [2]. Nonetheless, specific multiclass alternatives were also developed, e.g., by Kautz et al.: their multiclass performance score (MPS) was proven to be superior to eight performance metrics, including balanced accuracy, Cohen’s kappa, ACC (accuracy, correct classification rate), MCC (Matthews correlation coefficient), and F1 (see the abbreviations in Section 4.3 of this paper) on six real datasets, with the use of the *k*-nearest neighbor algorithm as the classifier [3].

An important factor that affects the performance of classification models is the balance (or imbalance) of the classes, i.e., the diversity in the number of samples belonging to each class. This is especially relevant in multiclass scenarios, where there can even be more than one minority class. The past decade has seen the development of several approaches to deal with class imbalance. Synthetic minority over sampling (SMOTE) [4] and its related methods are widely used to avoid biased results toward the majority class [5,6]. While oversampling can lead to overfitting in “classical” machine learning models, convolutional neural networks (CNNs) are less prone to overfitting [7]. Robust QSAR models can be developed for imbalanced high-throughput screening datasets using multiple undersampling as well [8]. Undersampling can also be achieved by clustering the majority class into the same number of clusters as the number of samples in the minority class: this approach proved to be efficient on small and large datasets as well [9]. The aggregated conformal prediction procedure has a promising potential for severely imbalanced datasets “to retrieve a large majority of active minority class compounds”, i.e., in a binary class situation [10]. An asymmetric entropy measure was recommended for classifying imbalanced data by Guermazi et al. They adapted the decision-tree algorithm to imbalanced situations, with a split criterion that discriminates the minority-class items on a binary-classification problem. They propose the ensemble approach for imbalanced learning [11,12].

Ensemble learning algorithms seem to be the solution for the classification of high-dimensional imbalanced data: König et al. have used mixtures of experts as a natural choice for the prediction of environmental toxicity [13]. According to Oza and Tumer, “classifier ensembles provide an extra degree of freedom in the classical bias/variance tradeoff, allowing solutions that would be difficult (if not impossible) to reach with only a single classifier” [14]. Fernandes et al. investigated twenty imbalanced multiclass datasets; they found that Ensemble of Classifiers based on MultiObjective Genetic Sampling for Imbalanced Classification (E-MOSAIC) provided the best predictive performance according to multiclass AUC (area under the receiver operating characteristic curve) and geometric mean [15]. Žuvela et al. also favor ensemble learning approaches for competing objectives and imbalanced situations [16]. Ensemble learning approaches constitute a current field of development, e.g., a new algorithm (HIBoost) applies a discount factor, which restricts the updating of weights, and hence the risk of overfitting is reduced [17]. Sets of simultaneous classifiers are also suitable to generate separation frontiers of classes naturally present in bioinformatics. The crucial step is to select metrics that measure performance of algorithms realistically [18].

Multiclass classification is employed for diverse applications in drug design and other fields; a few examples are listed here. Mandal and Jana have evaluated two machine learning algorithms, naïve Bayes (NB) classifier and *k*-nearest neighbors (*k*NN), to classify multiclass drug molecules. The *k*NN method shows higher accuracy and higher precision compared to NB. Furthermore, recall and F1 score of *k*NN are higher than that of NB [19]. Sokolova and Lapalme examined twenty-four performance measures for multiclass text classification from the point of view of invariance, identifying different sets of performance measures for the classification of human communication vs. documents [20]. (A measure is invariant if its value does not change when a confusion matrix changes; it can be beneficial or adverse, depending on the objective.) Idakwo et al. predicted androgen receptor activity (agonist, antagonist, inactive, and inconclusive) for 10,000 compounds, highlighting the use of deep neural networks (DNN), which significantly outperformed random forests (RF) according to four metrics (positive predictive value –PPV, true positive rate–TPR, F1, and area of PPV vs. TPR curve–AUprC) [21]. Multiple approaches for predictive QSAR models for classifying androgen receptor ligands were compared by Piir et al. using random forests [22].

Several authors have contributed with comparative studies for the better understanding of the various factors, choices, and alternatives in classification modeling. Chen et al. studied the effects of the decision threshold on three performance parameters (sensitivity, specificity, and concordance coefficient) for four classical classifiers: linear discriminant analysis (LDA), logistic regression, classification tree, and a weighted variant of *k*-nearest neighbor (*k*NN). A threshold of 0.5 can be used for balanced datasets; the change of decision threshold simply makes a tradeoff between the number of true positive and the number of true negative predictions, whereas the concordance coefficient does not vary much [23]. An ensemble classification approach is suggested for different class sizes in the case of binary classifier systems [24]. Two alternative thresholding strategies that maximize the geometric mean (GM) are suggested by Johnson and Khoshgoftaar [25]. Škuta et al. have studied QSAR-derived affinity fingerprints and established various AUC thresholds for different types of fingerprints [26]. Huang et al. have compared the performance of extreme learning machine (ELM) with that of least square support vector machine (LS-SVM) and proximal support vector machine (PSVM). Both LS-SVM and PSVM achieved suboptimal solutions, whereas ELM produced similar results for binary classification, but much better for the multiclass situation [27].

In spite of the widespread use of multiclass classification and the literature resources listed, a comparative study on the effects of dataset size and train/test split ratios is still lacking. Here, we address these questions on three case studies of absorption/distribution/metabolism/excretion (ADME) and toxicity-related data. Our approach involved a detailed, systematic analysis of variance (ANOVA) and multicriteria analyses to show the effect of the mentioned factors on the performance of multiclass classification. Additionally, we compared the applied 25 performance parameters in terms of their variances across different dataset sizes and split ratios. This work is an organic continuation of a previous study, where we compared machine learning algorithms and performance parameters for both two-class and multiclass problems [2].

## 2. Results and Discussion

We selected three case studies for studying the effects of dataset size and training/test split ratios on various machine learning classification models. To that end, modeling was repeated many times, with different versions of the starting datasets (leaving out different molecules from the majority group(s) to produce a balanced dataset), different numbers of samples (NS) and train/test split ratios (SR), and with five iterations for each combination of these parameters. After the iterative modeling process, 25 performance parameters were calculated: these constituted the columns of the input matrix for the statistical analyses (with the rows corresponding to the different parameter combinations). The performance parameters were calculated for the cross-validation and test validation as well. An example of the data structure is shown in Table 1. Principal component analysis (PCA) score plots were produced out for the visual description of the three datasets (see Figure S1). The original data matrices were compared with the balanced versions.

Factorial ANOVA was performed on the data matrix, where four factors were used: (i) the split ratios for the train/test splits (SR), (ii) the number of samples (NS) or dataset size, (iii) the applied machine learning algorithms (ML), and (iv) the performance parameters (PP). The factor values are summarized in Table 2.

### 2.1. Case Study 1

**Figure 1:**
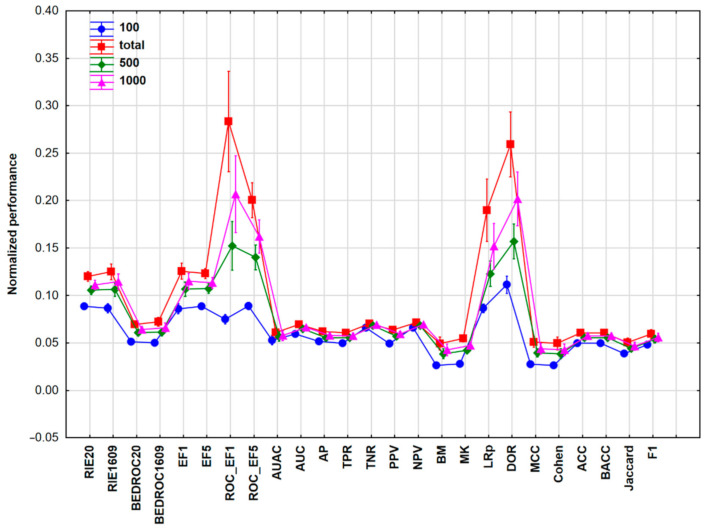
The average scaled values of the different performance parameters with the 95% confidence intervals for Case study 1. Normalization of performance parameters was necessary to be comparable. Number of samples (NS) is 100 (blue circles), 500 (green diamonds), 1000 (purple triangles), or total (2710, red squares). Abbreviations are explained in table from Section 4.3.

The first case study is a dataset of cytochrome P450 (CYP) inhibitors from the Pubchem Bioassay database (AID 1851), containing 2068 descriptors of 2710 molecules with reported inhibitory activities against either the CYP 2C9, or 3A4 isoenzyme, or both (thus, corresponding to a three-class classification). This case study therefore embodies a classification problem of selective-, medium-, and non-selective molecules, or toxic-, medium-, or nontoxic compounds, which is relevant in diverse subfields of drug design or the QSAR field. Factorial ANOVA was carried out on the input matrix of scaled performance parameters. Univariate tests showed that the ML, NS, and PP factors were significant in the analysis; however, the split ratios for the train/test splits (SR) did not have a large effect on the performance of the models. In Figure 1, the performance parameters combined with the dataset sizes (NS) are plotted based on their average scaled values.

Based on Figure 1, some performance parameters—such as AUC, AUAC (area under the accumulation curve), AP (average precision), TPR, TNR (true negative rate), PPV, or NPV (negative predictive value)—are not sensitive to changes in the dataset size. The most sensitive ones were the receiver operating characteristic (ROC) enrichment factors and the LRp (positive likelihood ratio) and DOR (diagnostic odds ratio) parameters, where higher performance values were detected with increasing dataset sizes.

**Figure 2:**
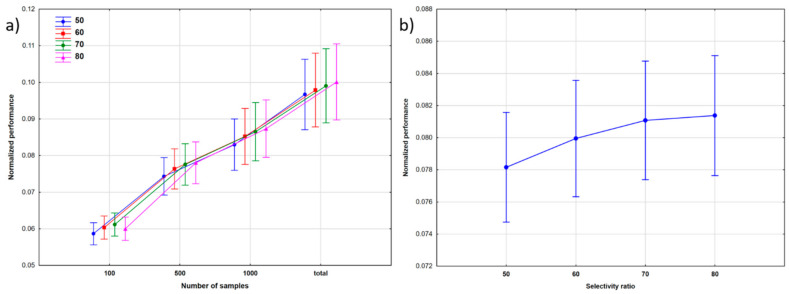
The effect of: (**a**) the number of samples (NS) at different split ratios, and (**b**) the split ratios (SR) on the performance of the models.

A combination of the NS and SR factors are plotted in Figure 2: here, the results show that the split ratios had a more significant effect on the modeling at bigger dataset sizes, and the overall performance of the models increased with the size of the dataset. The effect of the split ratio was not significant with 100 molecules in the data matrix, and generally, increasing the number of molecules in the training set from 50% to 80% conveyed only a small increase of the classification performance (Figure 2b). Since the comparison was dedicated to multiclass classification, it is not surprising that the differences between the split ratios were not significant at small sample sizes (where the model performances were far from satisfactory anyway), but the 70% and 80% split ratios clearly performed better for larger datasets. In these cases, the test sample was much smaller, but the performance of the models in test validations was actually not far from that in cross-validation. Tukey’s post hoc test was applied to establish the significance in the performances of different split ratios: the difference was significant only between 50% and 60%. We wanted to examine this effect in more detail; thus, we used sum of ranking differences (SRD) for the comparison of the split ratios (see later).

**Figure 3:**
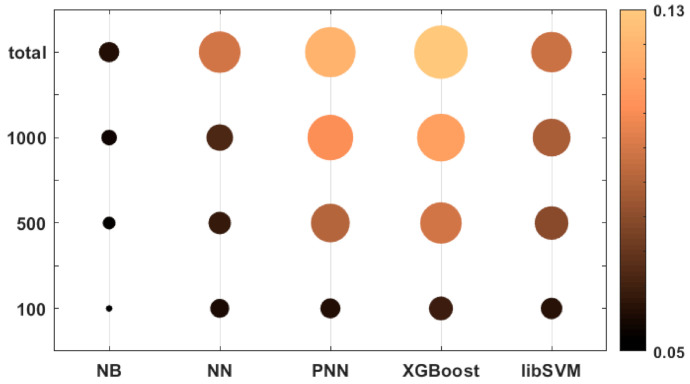
Bubble plot of the performances of the machine learning algorithms at different dataset sizes. Both the colors and the radii of the circles correspond to the average of the 25 normalized performance parameter values. (The bigger (and brighter) the better.).

In Figure 3, we present a bubble plot to visualize the differences between the dataset sizes and the applied machine learning algorithms. Both the colors and the radii of the circles correspond to the average of the 25 normalized performance parameter values.

Based on Figure 3, it is clear that the naïve Bayes (NB) algorithm had a lower performance compared to the other algorithms, even for bigger datasets. The most size-dependent methods were PNN (probabilistic neural network) and XGBoost, but these two algorithms could perform much better above 100 samples. XGBoost could achieve the best performance at the “total” level of the dataset size (2710 samples). The support vector machine (SVM) method, while performing slightly worse, was less size-dependent.

### 2.2. Case Study 2

**Figure 4:**
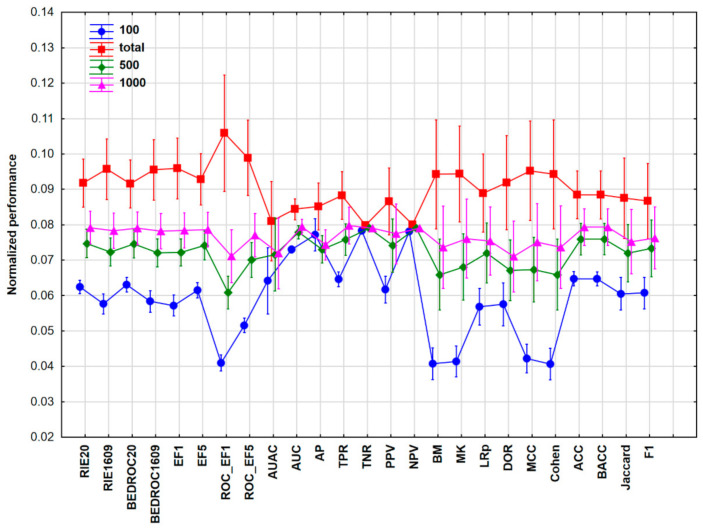
The average normalized values of the different performance parameters with the 95% confidence intervals for Case Study 2. Normalization of performance parameters was necessary to be comparable. Number of samples (NS) is 100 (blue circles), 500 (green diamonds), 1000 (purple triangles), or total (1542, red squares).

The same workflow was carried out for Case Study 2, which contained 2070 descriptors of 1542 molecules with measured acute oral toxicities (from the TOXNET database [28], downloadable here: https://www.epa.gov/chemical-research/toxicity-estimation-software-tool-test, last accessed on 18 February 2021) that were classified into six categories (from highly toxic to nontoxic [29]), thereby corresponding to a six-class classification into gradual categories. The 25 performance parameters were normalized and used for the ANOVA evaluation of both the cross-validation (CV) and test validation results. The univariate test showed that all of the four factors (PP, SR, NS, and ML) had a significant effect on the performance of the models. Figure 4 shows the average normalized values of the different performance parameters in combination with the NS factor (dataset size).

It can be noticed that the pattern of the results is quite similar to Case Study 1, which is not accidental: the dataset size had less effect on AUC, AP, TNR, and NPV. On the other hand, enrichment factors, bookmaker informedness (BM), markedness (MK), MCC, and Cohen’s kappa values are highly dependent on the size of the dataset (with higher performance values at larger sample sizes).

**Figure 5:**
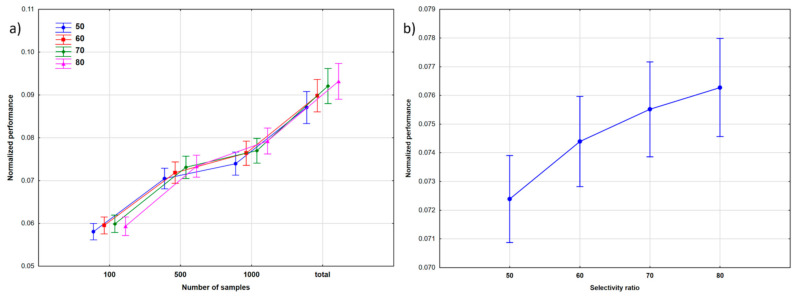
The effect of: (**a**) the number of samples (NS) at different split ratios, and (**b**) the split ratios (SR) on the performance of the models for Case Study 2.

A combination of the NS and SR factors is shown in Figure 5, similar to Case Study 1. Clearly, the performances improved with increasing dataset sizes. Moreover, the split ratios had bigger differences compared to Case Study 1, especially at bigger dataset sizes. This is further verified by Figure 5b and by Tukey’s post hoc test, which found significant differences between the performances at each of the four split ratios.

**Figure 6:**
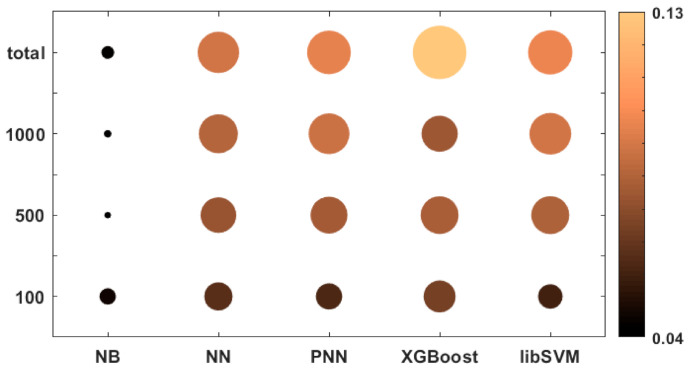
Bubble plot of the performances of the machine learning algorithms at different dataset sizes for Case Study 2. Both the colors and the radii of the circles correspond to the average of the 25 normalized performance parameter values. (The bigger (and brighter) the better.).

The effects of the dataset sizes upon the different machine learning algorithms were visualized in a bubble plot: Figure 6 shows that once again, the naïve Bayes (NB) method provided the worst performances, while the NN (multi-layer feed-forward of resilient backpropagation network), PNN, and libSVM (library for support vector machines) algorithms are very close to each other at every dataset size. Only XGBoost can be highlighted, as it performed better than the other algorithms in the case of the total number of samples (1542). Noticeably, each method, except for NB, performed better at bigger dataset sizes.

### 2.3. Case Study 3

In this case study, 1834 descriptors were calculated for 1734 different molecules with experimentally measured fraction unbound in plasma values (*f*_u,p_), based on the work of Watanabe et al. [30]. The dataset was categorized by the *f*_u,p_ values into three classes: low, medium, and high. The low range was assigned to molecules with *f*_u,p_ values below 0.05, the medium between 0.05 and 0.2, and the high above 0.2. The modeling workflow was the same as in the case of the two other case studies, and the 25 performance parameters were used for the ANOVA analysis after the normalization process for both CV and test validation.

**Figure 7:**
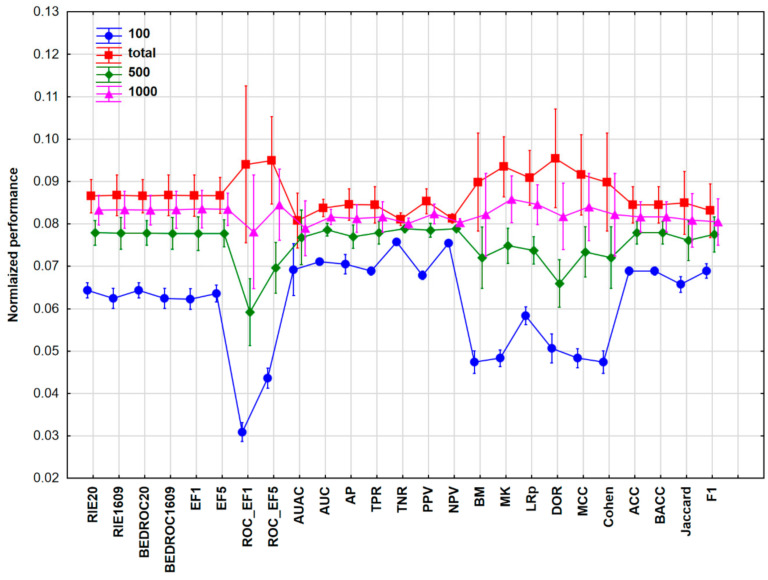
The average normalized values of the different performance parameters with 95% confidence intervals for Case Study 3. Normalization of performance parameters was necessary to be comparable. Number of samples (NS) is 100 (blue circles), 500 (green diamonds), 1000 (pink triangles), or total (1734, red squares).

All of the four factors—performance parameter, split ratio (SR), number of samples (NS), and machine learning algorithm (ML)—had significant effects on the modeling, based on the univariate test in the ANOVA evaluation. The average normalized performance parameter values turned out to be similar to the other cases. Figure 7 shows the result of the ANOVA analysis, with the combination of the performance parameters and the number of samples as factors.

In Figure 7, it is clearly shown that TNR and NPV were less sensitive to the dataset size, while the enrichment factors or DOR, MCC, and Cohen’s kappa depended more strongly on the dataset size.

**Figure 8:**
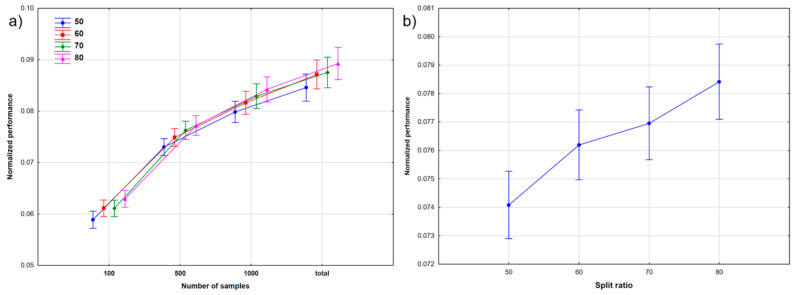
The effect of: (**a**) the number of samples (NS) at different split ratios, and (**b**) the split ratios (SR) on the performance of the models for Case Study 3.

The combination of the NS and SR factors was also evaluated and is shown in Figure 8. It is clear that the performance of the models increased with the dataset size. We can also observe a slight performance increase with increasing split ratios in Figure 8a, even for the smallest sample size (100). This increase can be seen in Figure 8b as well, with bigger differences between the different split ratios (as compared to Case Study 1); however, Tukey’s post hoc test still could not detect a significant difference between the 60 and 70 split ratios.

**Figure 9:**
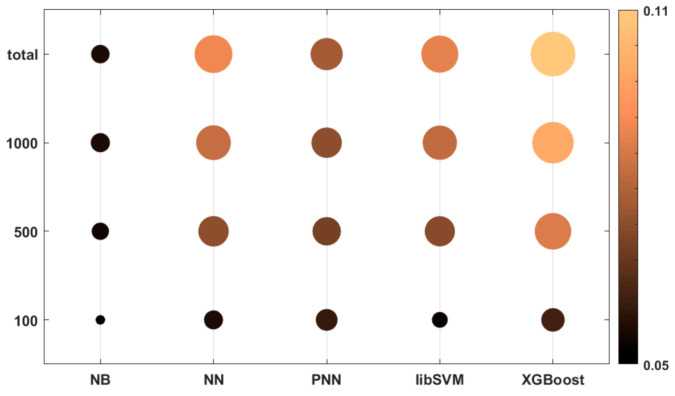
Bubble plot of the performances of the machine learning algorithms at different dataset sizes for Case Study 3. Both the colors and the radii of the circles correspond to the average of the 25 normalized performance parameter values. (The bigger (and brighter) the better.).

Figure 9 shows a bubble plot as the visualization of the ML and NS factors in combination. The results are very similar to the other two case studies: naïve Bayes (NB) models performed the worse in every case, NN and libSVM were moderately good, especially in the case of the total sample set (when all the 1734 molecules were used), and XGBoost had the best performance. In the smallest dataset size (100), the differences were much smaller between the methods. The findings are in good agreement with the previous case studies.

**Figure 10:**
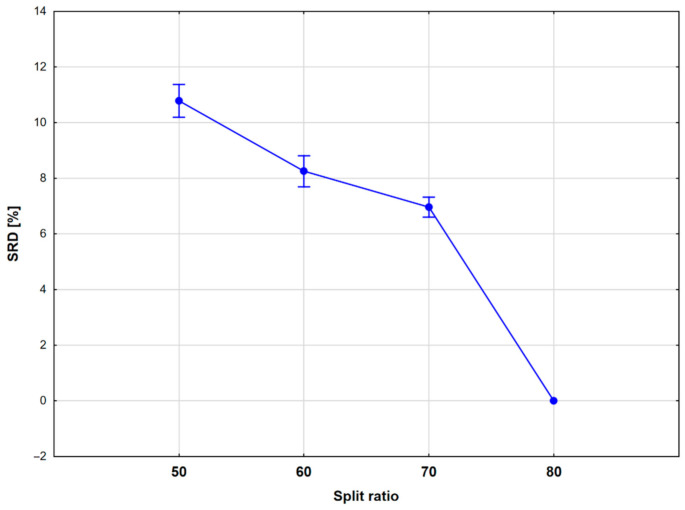
Sum of ranking differences (SRD) (%) values, plotted against the split ratio settings. Average values are marked with blue circles and 95% confidence intervals are also plotted.

Finally, SRD analysis was carried out to compare the different split ratios. Here, the three case studies were merged, and the performance parameters were normalized together for the three case studies. In the input matrix, the performance metrics (25) were in rows and the split ratios (four) in the columns. Row-maximum was used as the reference for the analysis (corresponding to an ideal model that maximizes each performance parameter), with five-fold cross-validation. All of the four split ratios provided better results than random ranking. The cross-validated SRD results were evaluated with a one-way ANOVA, where the only factor was the split ratio (SR). The ANOVA results are presented in Figure 10: the univariate test identified significant differences between each of the split ratios; thus, the SRD results provided a more sensitive analysis of the mentioned factor. The split ratios were farther from each other in terms of the SRD values, but still the 80% split ratio achieved the best result (smallest SRD value) by far: in fact, it was identical to the reference values, meaning that the performance was better than the other settings according to each performance parameter.

As a summary of our results, we could show that the performance of the multiclass classification models can greatly depend on all the examined parameters. As for performance parameters, there was a smaller group (such as AUC, AP, TNR and NPV), which was relatively independent from the dataset sizes. The machine learning algorithms also differed from each other in the sense of performance: in comparison with current alternatives, the naïve Bayes algorithm was not a good option for multiclass modeling. On the other hand, XGBoost was a viable one, especially for large sample sizes. The ANOVA results showed that the split ratios had a stronger effect on performance at larger dataset sizes. Moreover, the SRD analysis was sensitive enough to find smaller differences in the dataset, and we could select the most prominent factor combinations to achieve better predictive models. Based on our findings, we can suggest the use of the 80%/20% training/test split ratio, especially for larger datasets, to provide enough training samples even for multiclass classification.

## 3. Discussion

Several machine learning algorithms were evaluated by Valsecchi et al. [31], comparing multitask deep (FFNL3) and shallow (FFNL1) neural networks with single-task benchmark models such as *n*-nearest neighbor (N3), *k*-nearest neighbor (*k*NN), naïve Bayes (NB), and random forest (RF) techniques, evaluated with three performance parameters: sensitivity, specificity, and non-error rate (or accuracy). The multitask scenario presents an alternative approach to the multiclass situation in our Case Study 1, i.e., when the classification is based on multiple properties that are—theoretically—independent (here, inhibitory activities of two isoenzymes), with multitask models providing separate predictions for each property. In the work of Valsecchi et al., no approach outperformed the others consistently: task-specific differences were found, but in general, less represented classes are better described using FFNL3. Perhaps their most striking conclusion is that single task models might outperform the more complex deep learning algorithms, e.g., surprisingly, naïve Bayes is superior to FFNL3 in certain situations. There is no doubt that N3 is the best, while NB is the worst single-task algorithm, but even this limited number of performance parameters reveals the dataset-size dependence. Our present work clearly manifests the overall inferiority of NB compared to the other machine learning algorithms examined and highlights XGBoost as the best option among those that were considered.

The impact of class imbalance on binary classification was studied by Luque et al. [32]. Ten performance metrics were evaluated based on binary confusion matrices, with the authors favoring the Matthews correlation coefficient for error consideration. For imbalanced data, the performance metrics can be distributed into three clusters considering the bias measure: zero (TPR, TNR, BM, and the geometric mean of sensitivity and specificity, GM), medium (ACC, MCC, MK), and high bias (PPV, NPV, and F1). While we worked with balanced datasets, it is interesting to observe that some of the low-bias measures—BM, MK, and MCC—exhibit a strong dataset size dependence, see Figure 4 (Case Studies 2 and 3, high variance), when a complex interplay of various factors is considered: machine learning algorithms, training/test split ratios, number of compounds were varied.

Lin and Chen addressed the fundamental issues of class-imbalanced classification: imbalance ratio, small disjoints, overlap complexity, lack of data, and feature selection [33]. They claim that the SVM-ensemble classifier performs the best when the class imbalance is not too severe. This is in agreement with our work: here, SVM is identified as a competitive approach (although still somewhat inferior to XGBoost).

To summarize, our 25 performance measures were exhaustive and provided a more sophisticated consensus about performance. The number of samples and the train/test split ratio exerted a significant effect on multiclass classification performance. Of course, all comparative studies are influenced by the specific structure of the datasets, but overall tendencies and optimal solutions can be identified.

## 4. Materials and Methods

### 4.1. Datasets

Three datasets (Case Studies 1, 2, and 3) from the ADME and toxicity area were selected carefully. The first dataset corresponded to a case study of selectivity: here, cytochrome P450 inhibitors were classified based on their inhibitory activity against either the 2C9 or 3A4 isoenzyme, or both (presenting a three-class classification scenario). The datasets assembled 4570 molecules with measured inhibitory levels on cytochrome P450 2C9 and 3A4 isoenzymes from the publicly available PubChem Bioassay (NCBI) database (AID1851) [34]. Molecules without SMILES codes, duplicates, and inconclusive class memberships were excluded from the dataset. Classical 1D and 2D descriptors were calculated by Dragon 7.0 (Kode Cheminformatics, Pisa, Italy) [35] with an intercorrelation limit of 0.997 [36]. Near-constant descriptors were also omitted. The dataset was balanced in order to improve the performance of the models [2]. Balanced design is important especially in the case of multiclass classifications. Finally, the dataset consisted of 2710 molecules and 2068 descriptors.

The second case study was an acute oral toxicity dataset, corresponding to a case study of gradual levels: the dataset contained molecules with 50% oral lethal dose (LD50) values measured on rats (from the TOXNET database, downloadable from: https://www.epa.gov/chemical-research/toxicity-estimation-software-tool-test, accessed on February 18, 2021) [28], which were categorized into six toxicity classes, according to the Globally Harmonized System of Classification and Labelling of Chemicals (GHS) [29]. After the data curation process and balancing, 1542 molecules remained in total. The descriptor calculation was carried out in the same way as for the first case study. Finally, the dataset consisted of 1542 molecules and 2070 descriptors.

The third case study was the fraction unbound in plasma (*f*_u,p_) dataset, which is an important parameter of drug efficacy [30]. The experimental values were categorized into three classes: low, medium, and high. The dataset was balanced as in the other two cases. After balancing, 1734 molecules in total remained, out of 2319. After the same descriptor calculation process as in the previous cases, 1834 descriptors were applied for the modeling process.

It is worth to note that Case Study 1 exhibited small variances, whereas Case Studies 2 and 3 were of high variance. Hopefully, joint conclusions apply generally.

### 4.2. Machine Learning Algorithms

Five different machine learning algorithms were apt for model building: XGBoost, RPropMLP, PNN, libSVM, and naïve Bayes (NB). XGBoost is a well-known tree-based algorithm, which is the “upgraded” form of the boosted tree method [37]. RPropMLP is the multi-layer feed-forward of resilient backpropagation network, which is a variant of artificial neural networks. Here, local adaptation of the weight updates was included, which is similar to the error function [38]. Throughout this article, RPropMLP is shortened to NN. The other neural network based algorithm was PNN [39], which is a probabilistic neural network with dynamic decay adjustment, and it generates rules with a high-dimensional Gaussian function based on the numerical data. LibSVM is a support vector machine (SVM) algorithm [40] implemented in the Weka package and the KNIME platform, with sequential minimization and a radial basis function. The naïve Bayes classifier was also applied, which is a simple and well-known probabilistic classifier based on Bayes’ theorem and the assumption of the independence of all attributes [2,41].

It is important to note that we had no intention to produce optimized models (the models reported here can be outperformed easily) but rather to make the modeling reproducible, comparable, and consistent for the evaluation of the different modeling parameters in the three case studies. Thus, for the sake of being comparable across the five algorithms, there were no additional feature selection and parameter optimization steps carried out. For all five algorithms, the default parameter settings were used for model building, as implemented in KNIME Analytics Platform v4.2.0 (KNIME AG, Zurich, Switzerland).

### 4.3. Modeling

**Figure 11:**
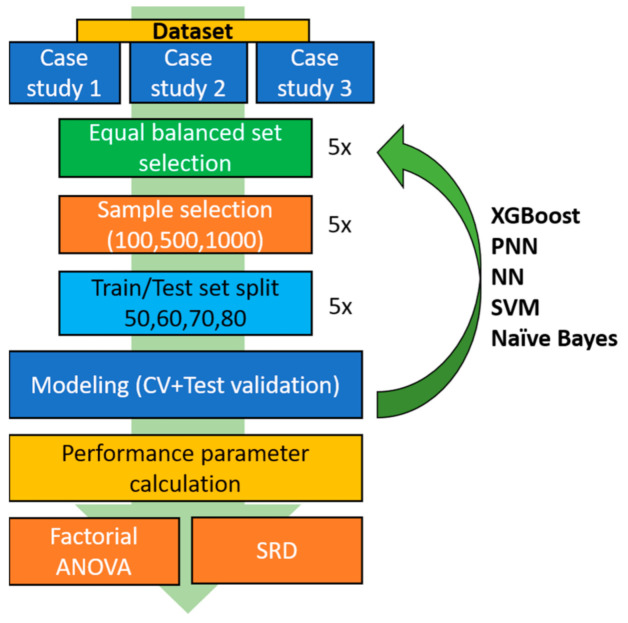
Workflow of the modeling process: for each case study, balanced set selection was performed in five repetitions (to account for all available molecules during modeling). Sample selection was then carried out in five repetitions for three samples sizes, and train/test splitting was performed in five repetitions for four split ratios, resulting in altogether 125 models for each combination of settings. Then, 25 performance parameters were calculated for each model (both for cross-validation and test validation), which were used to compare the models with factorial ANOVA and sum of ranking differences (SRD).

A full modeling workflow was assembled in KNIME Analytics Platform 4.2.0 (Figure 11). The modeling workflow had six major steps from descriptor generation to the analysis of the performance parameters. First, the descriptor set was standardized with *z*-scores. Then, an equal balance sampling was carried out, where the number of the molecules in the smallest group was kept from the other groups as well. In this process, an iterative random selection of the molecules was implemented with five iterations. In the third step, we randomly selected 100, 500, or 1000 molecules from the dataset (or kept all of them) in five iterations (number of samples, NS), except—naturally—when the total number of samples were kept. Next, the selected molecules were split to training and test sets, keeping 50%, 60%, 70%, or 80% of molecules in the training set (split ratio, SR); this was also repeated five times for each split ratio. The actual modeling was started for each of the resulting combinations and iterations, where a five-fold cross-validation on the training set (with a fixed random seed to ensure reproducibility) and a test validation on the test part was carried out. Altogether, 125 different models were developed for each dataset size and split ratio combination. Finally, 25 performance parameters were calculated for every model, which were normalized and averaged for each combination before the statistical analysis part of the results.

The applied modeling workflow can be found in the Supplementary Materials. Modeling was carried out on a PC with Intel-Core i7 CPU, 3.40 GHz, and 16 GB RAM (with Windows 7 OS). The compute time of a full workflow for one algorithm (which included 1500 models) was 16.9 h in average. The fastest algorithm was the naïve Bayes, while the calculation was more time-consuming for the libSVM and XGBoost algorithms.

The applied 25 performance parameters are summarized in Table 3; their definitions are included in our recent work [2].

### 4.4. Statistical Analysis

The results contained the calculated performance parameters for each factor combination (cross-validation and test validation as well). Euclidean normalization was applied for the performance parameters. The results for Case Studies 1 and 2 were evaluated with factorial variance analysis (ANOVA) and sum of ranking differences (SRD) [42]. Factorial ANOVA analysis compares the averages of the different groups based on several categorical factors. In our case, the analysis contained the following factors: (i) the split ratios for the train/test splits (SR), four levels; (ii) the number of samples (size of the dataset) (NS), four levels; (iii) the applied machine learning algorithms (ML), five levels, and (iv) the performance parameters (PP), 25 levels. Tukey’s post hoc test was used to establish whether the differences between the groups were statistically significant. SRD was used when the results of ANOVA with post hoc analysis were not sufficient to explore the differences between the groups. ANOVA analysis was performed in Statistica 13.5 software (TIBCO Software Inc., Palo Alto, CA, USA).

In the case of SRD analysis, the columns (variables) of the dataset were compared to a reference/gold standard. In our case, the performance parameters were in the rows; thus, the row-maximum values were used as reference, corresponding to an ideal model that maximizes each performance parameter. It means that the best method/selectivity ratio etc. should be the closest one to the vector defined by the maximum values of the performance parameters. SRD rank transformed the values in each column and in the reference as well, and then the ranks were compared between each column and the reference. Finally, the ranking differences were summed up for every column, which gave us the SRD value for each variable (column). SRD analysis was validated in two steps: a randomization test and five- to ten-fold cross-validation. A detailed summary of the SRD process (with animation) can be found in the supplementary material of our earlier work [43].

## 5. Conclusions and Outlook

The present work was dedicated to study the effects of dataset size and training/test split ratios on the performance of multiclass classification. Classification performances were quantified by 25 performance parameters whose averages (after normalization) constituted the basis for comparing different machine learning methods with different settings. The figures show the main results in a visually appealing way. The number of samples (compounds) and split ratio of training and test sets exerted a significant effect on multiclass classification performance. XGBoost provided the best overall performance, while the standard naïve Bayes classifier was outperformed by all the other models in all the situations studied.

As always, the performances depended on the data structure of the datasets investigated. Still, the presented methodology can provide a more optimal factor combination than any of the factors separately. A multicriteria analysis method called sum of ranking differences (SRD) can easily be performed to eliminate suboptimal solutions. In addition to the effects of dataset size and split ratios, we also compared the applied 25 performance parameters in terms of size-dependence, identifying several (especially BM, MK, MCC, Cohen’s kappa, and ROC enrichment factors) that were more sensitive to differences in the size of the input dataset.

## Figures and Tables

**Table 1:** The structure of the summarized results of modeling. (ML: Machine learning algorithms, SR: train/test split ratio, expressed as the percentage of compounds assigned to the training set, NS: number of samples, NB: naïve Bayes, CV: cross-validation).

ML	SR	NS	Split	Performance Parameters
NB	50	100	CV	x_1,1_, …, x_1,j_
NB	60	100	CV	
NB	70	100	CV	
NB	80	100	CV	
NB	50	500	CV	
…	…	…	…	…
XGBoost	80	total	test	x_i,1_, …, x_i,j_

**Table 2:** Labels and levels of the different factors.

Code	Name	Values/Categories/Levels
PP	Performance parameters	25 different parameters (see table from Section 4.3)
ML	Machine learning algorithm	XGBoost, naïve Bayes, support vector machine (SVM), NN (multi-layer feed-forward of resilient backpropagation network or RPropMLP) and probabilistic neural network (PNN)
NS	Number of samples, dataset size	100, 500, 1000, and total (all samples of the balanced dataset)
SR	Split ratios for the train/test splits	50, 60, 70, 80

**Table 3:** Summary of the applied performance parameters.

Abbreviation	Name
RIE20	Robust initial enhancement with α = 20
RIE1609	Robust initial enhancement with α = 160.9
BEDROC20	Boltzmann-enhanced discrimination of receiver operating characteristic with α = 20
BEDROC1609	Boltzmann-enhanced discrimination of receiver operating characteristic with α = 160.9
EF1	Enrichment factor at 1%
EF5	Enrichment factor at 5%
ROC_EF1	Receiver operating characteristic (ROC) enrichment at 1%
ROC_EF5	ROC enrichment at 5%
AUAC	Area under the accumulation curve
AUC	Area under the ROC curve
AP	Average precision
TPR	True positive rate, sensitivity, recall
TNR	True negative rate, specificity, selectivity
PPV	Positive predictive value, precision
NPV	Negative predictive value
BM	Bookmaker informedness
MK	Markedness
LRp	Positive likelihood ratio
DOR	Diagnostic odds ratio
MCC	Matthews correlation coefficient
Cohen	Cohen’s kappa
ACC	Accuracy, correct classification rate (CC)
BACC	Balanced accuracy
Jaccard	Jaccard score
F1	F1 score, F measure

## Data Availability

The core datasets are available online at the referred sites; calculated data are available from the authors upon request.

## Supplementary Materials

The following are available online: The applied KNIME workflow is provided and the PCA plots can be found as Figure S1.
