# Supplementary material for: Effect of Dataset Size and Train/Test Split Ratios in QSAR/QSPR Multiclass Classification
Source: Molecules. 2021 Feb 19;26(4):1111. doi: 10.3390/molecules26041111 (PMC7922354; doi:10.3390/molecules26041111)
Supplement: Supplementary file 1 [file molecules-26-01111-s001.pdf]

Supplementary Materials

Effect of Dataset Size and Train/Test Split Ratios in QSAR/QSPR Multiclass Classification

Anita Rácz [1], Dávid Bajusz [2] and Károly Héberger [1,*]

(**a**)

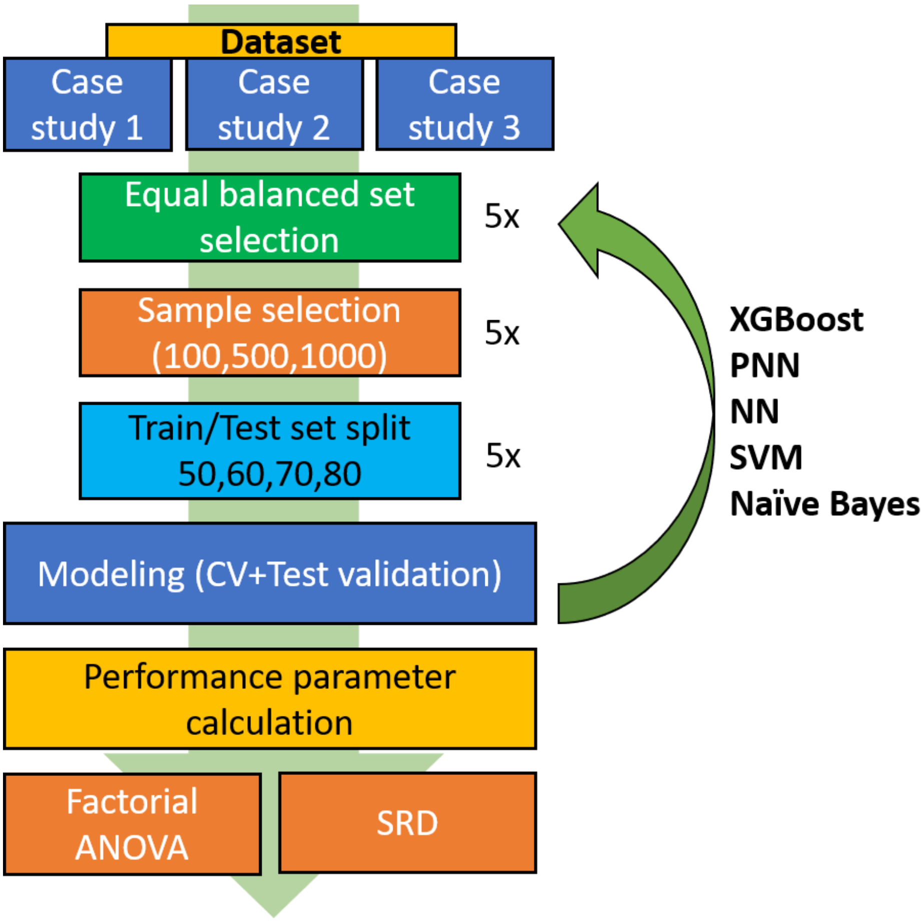

(**b**)

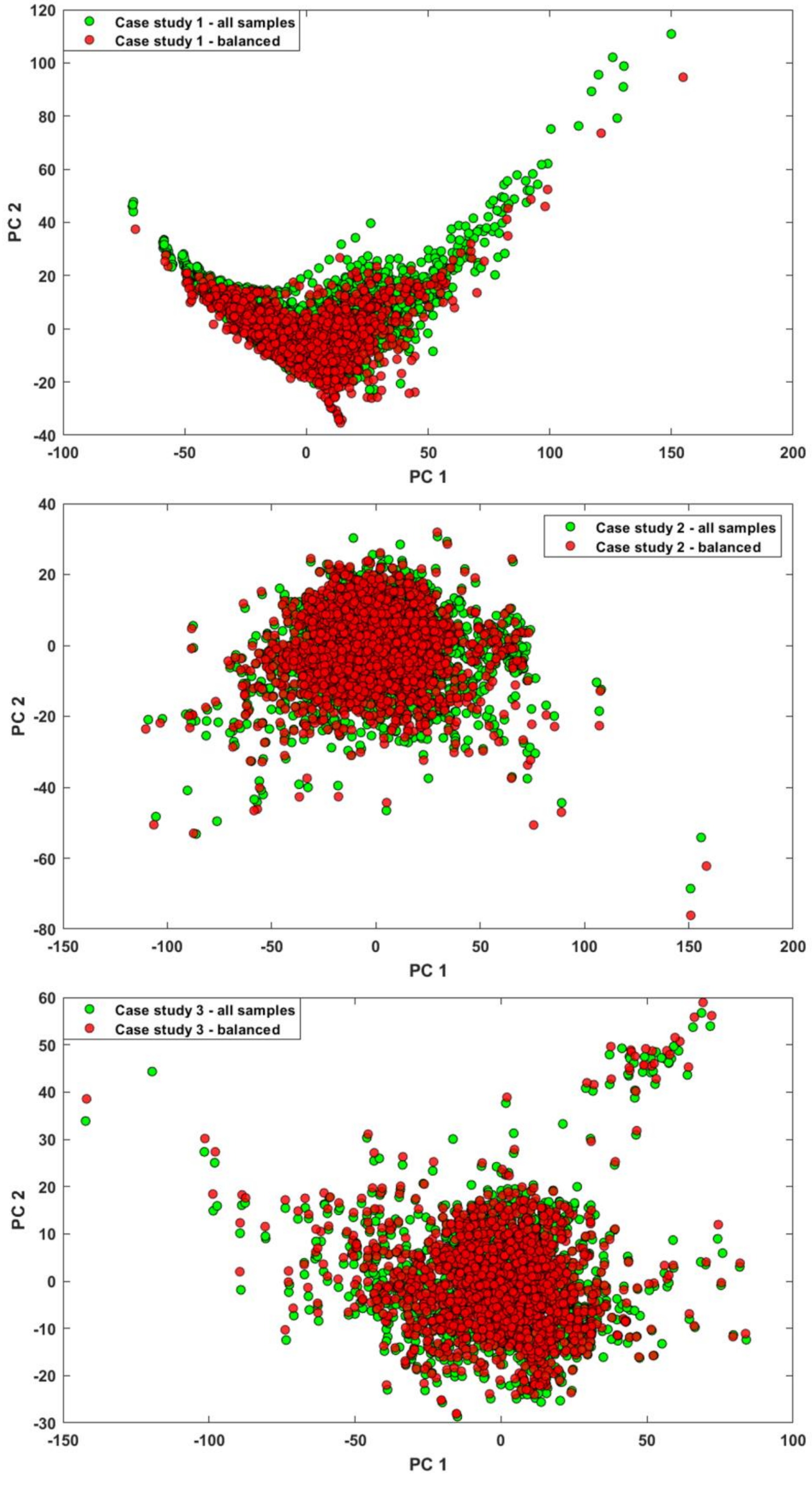

Figure S1. The applied modeling workflow (**a**). Principal component analysis (PCA) score plots (**b**).
